# A Full Systematic Review on the Effects of Cognitive Behavioural Therapy for Mental Health Symptoms in Child Refugees

**DOI:** 10.1007/s10903-021-01151-5

**Published:** 2021-02-15

**Authors:** Katie Lawton, Angela Spencer

**Affiliations:** 1 St Helens and Knowsley Teaching Hospitals NHS Trust, 474, Kings Road, Stretford, Manchester, M32 8QW UK; 2 University of Manchester, Oxford Road, Manchester, M13 9PL UK

**Keywords:** Refugee, Asylum-seeker, Child, Cognitive-behavioural therapy, Mental health, Psychological therapy

## Abstract

Global conflict in 2019 created record numbers of displaced children. These children have experienced multiple traumas and subsequently suffer high levels of mental health symptoms. Cognitive-behavioural therapy (CBT) is commonly used for post-traumatic stress disorder (PTSD), depression and anxiety, however the current evidence-base of CBT in child refugees is sparse, with mixed results. This study aimed to assess the effects of CBT on symptoms of PTSD, depression and anxiety in child refugees/AS. Ethics were reviewed and granted by the University of Manchester ethics committee. Medline, Embase, Cochrane, PsycINFO and CINAHL were systematically searched. Studies were included if CBT was delivered to refugee/AS children with pre and post-intervention measures of symptoms. Sixteen studies fulfilled criteria. In all studies, mental health symptom scores post-intervention had reduced, suggesting an improvement in mental health following CBT. This reduction was statistically significant in twelve studies (p < 0.001–0.5), clinically significant in eight studies and maintained at follow-up periods. No adverse effects of CBT were identified. This is the first systematic review to focus solely on CBT in child refugee populations, with unanimously positive results. Its use is cautiously recommended, however the need for more methodologically rigorous studies in this population is highlighted.

## Introduction

### Background

Levels of displacement experienced globally in 2019 were the highest ever on record, with an estimated 70.8 million people forced to flee their homes due to conflict and persecution (United Nations High Commissioner for Refugees (1). This has created more than 29 million refugees and asylum seekers worldwide, half of whom are under the age of 18 (1).

The 1951 United Nations Convention Relating to the Status of Refugees defined a refugee as.

“A person who owing to a well-founded fear of being persecuted for reasons of race, religion, nationality, membership of a particular social group or political opinion, is outside the country of his nationality and is unable or, owing to such fear, is unwilling to avail himself of the protection of that country; or who, not having a nationality and being outside the country of his former habitual residence as a result of such events, is unable or, owing to such fear, is unwilling to return to it.” (2). An asylum seeker (AS) is “a person who has left their country of origin and formally applied for asylum in another country but whose application has not yet been concluded” (3).

Child refugees are frequently subject to multiple stressors and traumas (4), which are conventionally described in terms of “phases” of the journey; pre-flight, flight and resettlement. Pre-flight includes the initial traumas they were escaping from, and the upheaval of moving. Flight includes unsafe transit and living in transitional placements such as refugee camps. Resettlement stressors can just be as traumatising as the initial stressors for children (5), and include time spent in detention centres, uncertainty about the future, poverty and cultural shock (6). Subsequently up to 80% of refugee children have depression, PTSD and anxiety (7–13). This rate is more than twice that of U.S. adolescents (14). Furthermore, refugee children with mental health symptoms are at greater risk of physical health symptoms (15), poorer educational attainment (16) and correlate with acculturalisation issues (17). Prompt identification of such mental health problems helps to reduce self-harm and suicide (18).

Despite their high levels of mental health morbidity, refugee children are under-represented in mental health services (19), with 49% of mental health symptoms unmet (20). This is despite evidence that treatment can reduce the mental health symptoms of refugees (21). Research has highlighted linguistic and socio-cultural barriers, difficulties navigating services, and financial and transport constraints as barriers to service access (22–25).

Cognitive-behavioural therapy (CBT) is a psychological therapy commonly used in the treatment of depression, PTSD and anxiety (26). The Royal College of Psychiatrists define CBT as a verbal therapy which considers how an individual appraises a situation, how these thoughts affect their emotions and behaviour and subsequently how the behaviour and emotions feed-back into cognitions (27). In CBT the therapist and client work collaboratively in changing the client's maladaptive thinking and behavioural patterns, releasing them from vicious cycles of negativity and learning practical skills to move forwards. Trauma-focused CBT is a subset of CBT that is specifically adapted for PTSD symptoms.

CBT is considered one of the most efficacious and evidence-based interventions for PTSD in traumatised non-refugee children, particularly trauma-focused CBT (28–30). CBT randomised controlled trials (RCTs) conducted on war-traumatised non-displaced children demonstrated successful reductions in PTSD, anxiety and depression symptoms, with effect sizes ranging from medium to large (Cohen’s d = 0.76–1.24), (31–33). Morina et al. (2017) conducted a meta-analysis of CBT on children living within war and reported pre/post CBT medium effect sizes for PTSD symptoms (Hedge’s g = 1.15), and small effect sizes of CBT on depressive symptoms (Hedge’s g = 0.25–0.3) (34).

Logically it follows that CBT is an appropriate treatment option for refugee children. Several systematic reviews have compared differing psychological therapies for this population. Tyrer and Fazel (2014) included eight primary CBT studies in their review of fourteen psychological therapies and found that mental health symptoms of depression, PTSD and anxiety were significantly reduced in all studies, with medium to large Cohen’s d effect sizes where calculated (d = 0.64–0.93) (35). Sullivan and Simonson (2016) included four primary CBT studies in their systematic review of thirteen psychological therapies and found that mental health symptoms were significantly reduced in all four studies, although no effect sizes were given (36). Both studies noted that CBT interventions showed the most consistent outcomes and highlighted its feasibility in school settings.

In contrast, Nocon et al. (2017) reviewed school-based therapies for depression and anxiety in child refugees and found small or insignificant effect sizes (37). However, the authors commented that “CBT showed the most promising results that need further replication” (37). All three systematic reviews called for further research to clarify findings relating to CBT.

This systematic review answers those calls and is the first to have a narrow research focus on the effectiveness of CBT in child refugee populations.

## Objectives

Objective 1.) To assess the effects of CBT on PTSD, anxiety and depression in child refugees/AS.

Objective 2.) To assess the accessibility and acceptability of CBT for child refugees/AS.

## Methods

### Search Strategy

Review methods were conducted in accordance with the Preferred Reporting Items for Systematic Reviews and Meta-Analyses (PRISMA) guidelines (38).

Ethics were reviewed and approved from the University of Manchester ethics review committee in September 2018. The review was registered with PROSPERO in January 2019.

### Criteria for Inclusion of Studies

#### Population

Participants must be refugees/AS aged 2–18.

#### Intervention

Studies were included if CBT was delivered as a treatment modality, in all intervention settings and delivered by any individual. The description of the CBT used needed to align with the Royal College of sec3Psychiatry’s definition of CBT described in the introduction Sect. (57), however all subtypes of CBT were included. Studies which used CBT in combination with other treatment methods were excluded as it would not be possible to extract the outcomes of CBT alone. Only studies which delivered a programme of CBT were included, rather than a singular session. The CBT could be delivered individually or as group-therapy.

#### Comparison

Studies were included if they involved either a within-subject or a between-subjects comparison of PTSD, anxiety or depression. Equivalence studies were included if it was possible to extract the pre and post-outcome measures for the population receiving CBT.

#### Outcomes

The primary outcome of interest for objective one was any change in PTSD, anxiety or depression pre and post-CBT, measured using a validated scale. Any adverse effects were also recorded.

The primary outcome of objective two was any recorded expressions of acceptability and feasibility made by the children or those delivering the intervention. Due to time constraints, only studies included for objective one will be analysed for these.

#### Study Types

Only primary research studies were included. This incorporated randomised controlled trials and observational studies with an interventional component and measurable outcomes. Preliminary database searches have confirmed a paucity of large-scale studies on this topic, and therefore case-series and case-studies were also included. Only studies reported in the English language were included.

### Search Terms

**Fig. 1:**
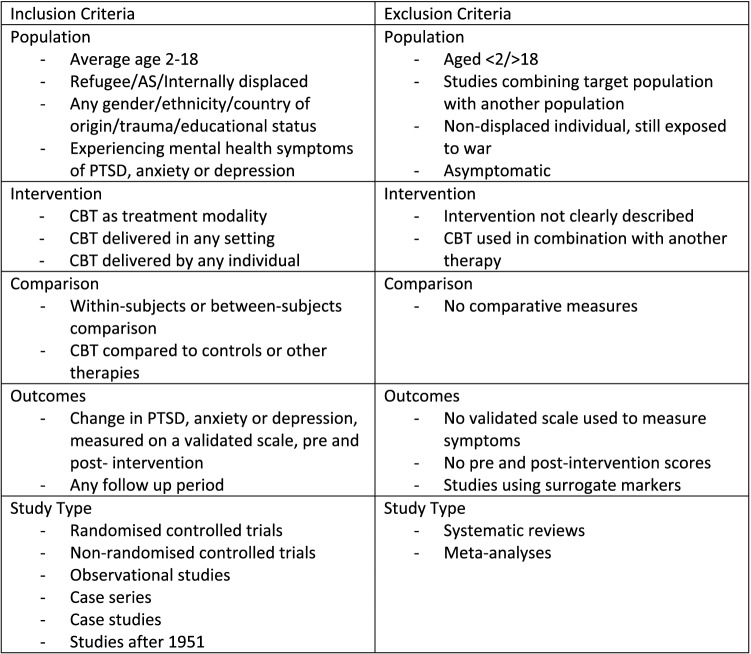
Inclusion and Exclusion Criteria

The search strategy was developed in consultation with an academic librarian, and included key terms and synonyms associated with (1) adolescent/child, AND (2) refugees/asylum seekers/ “displaced by war”, AND (3) cognitive therapy/ psychological adaptation (see Appendix A for full search strategies used) (Fig. 1)

In April 2020 five data-bases were systematically searched: Medline, Embase, Cochrane, PsycINFO and CINAHL. Hand searching was conducted to identify any key grey literature in the area.

## Results

**Fig. 2:**
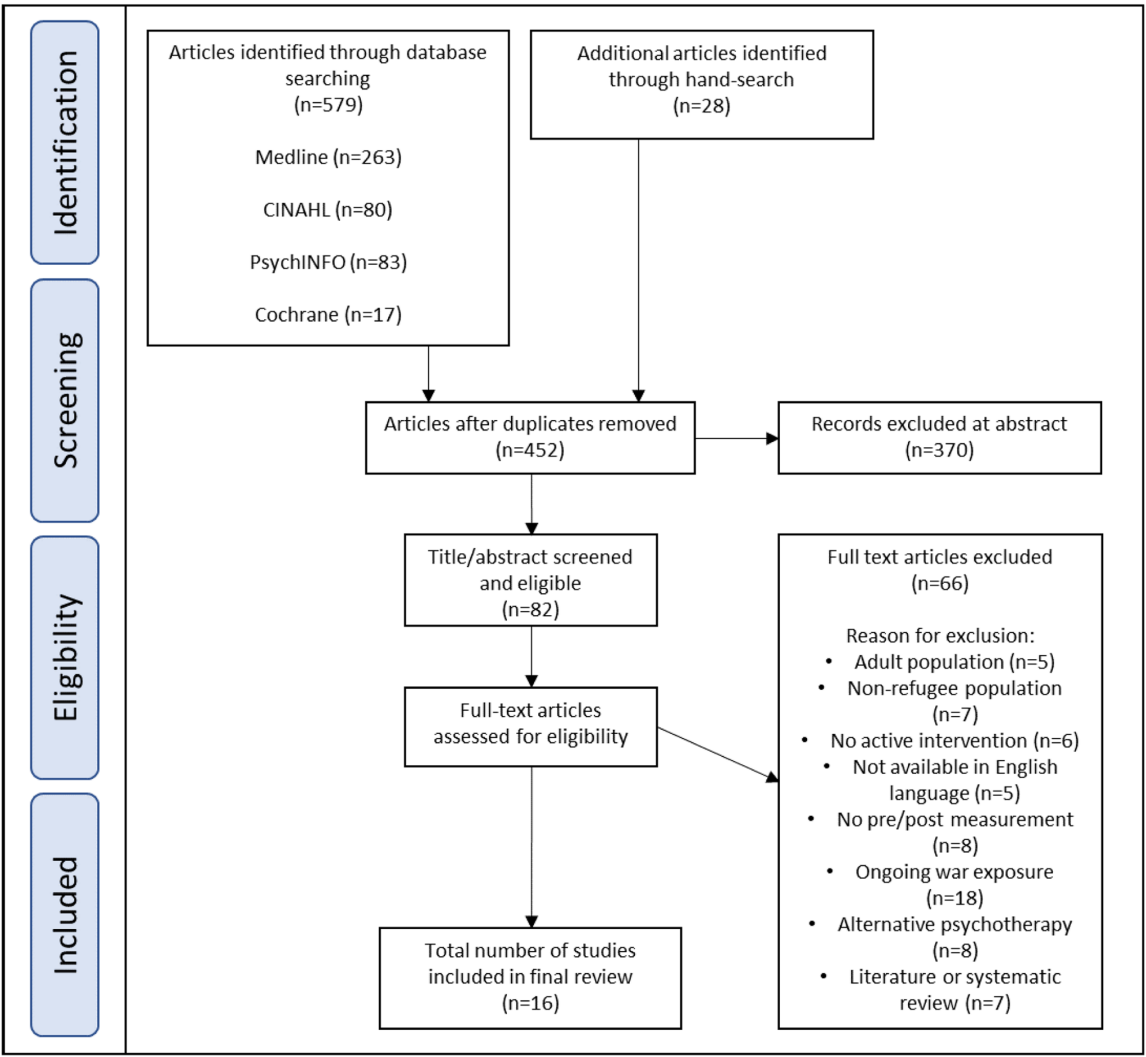
PRISMA flow-diagram

The details of literature search and study selection procedure are outlined in the PRISMA flow diagram (Fig. 2). A total of 579 studies were identified via the electronic database search, and an additional 28 articles via hand-searching and the snowballing technique, whereby studies of interest were identified from the reference list of highly relevant articles. The abstracts of 452 studies were reviewed by two authors (KL and AS), and 82 studies were eligible for full-text screen. Full texts were screened by both authors and 16 studies were found to meet the inclusion criteria.

### Data Extraction

**Fig. 3:**
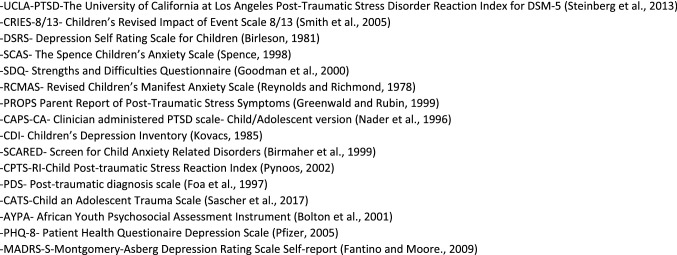
Abbreviations for outcome measures

To standardise the process, a data extraction table was created based upon the Cochrane data extraction table (38). No missing information was identified and thus contact with authors was unnecessary. Abbreviations for the outcome measures will be used from this point onwards, below in Fig. 3 is a list of the measures used.

## Methodological Quality

**Fig. 4:**
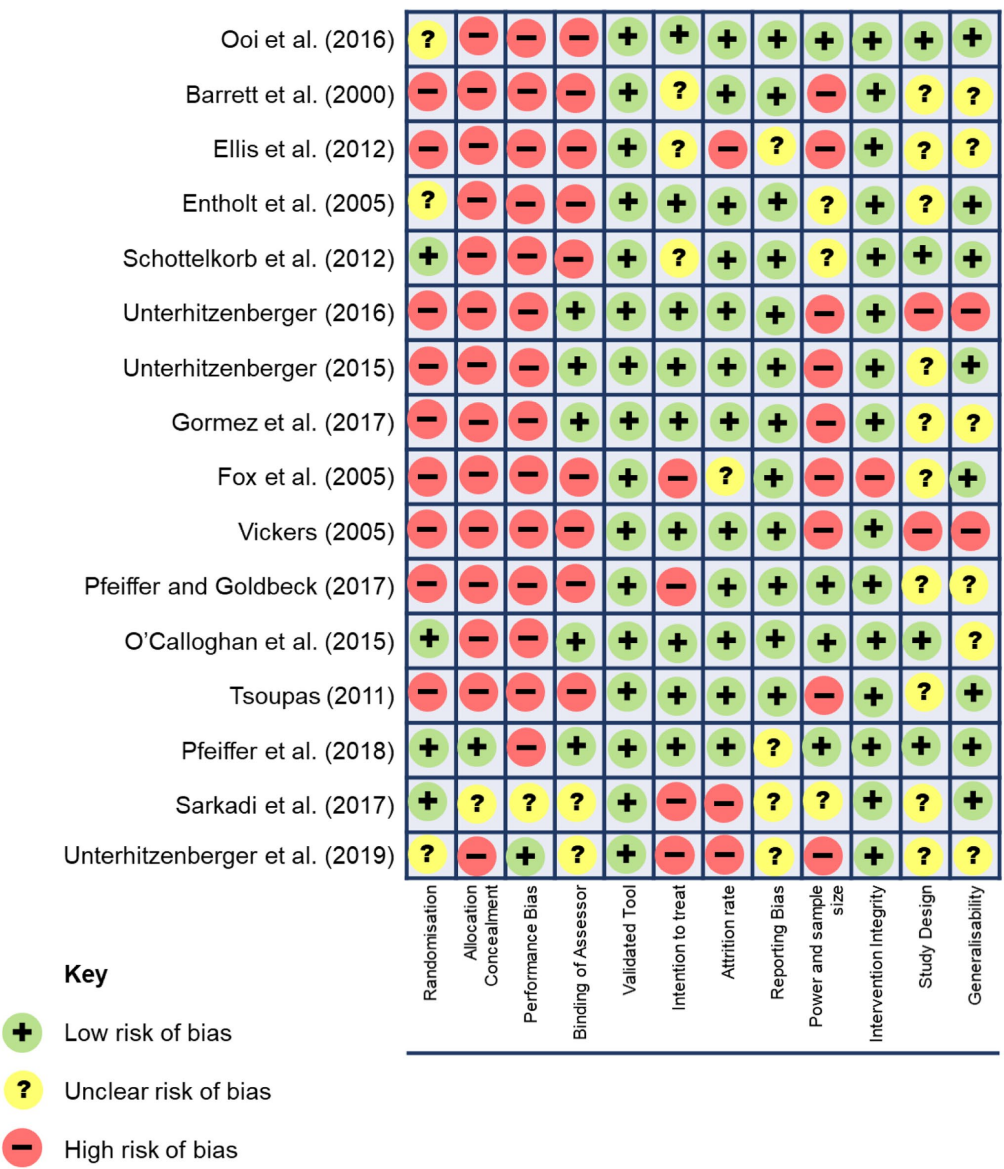
Risk of Bias in included studies

The Cochrane collaboration recommend against the use of numerical scales to asses bias, due to the “lack of empirical evidence”, instead recommending rating bias as “low” “high” or “uncertain” risk against several domains (38). Figure 4 summarises the risk of bias assessment for this review.

The nature of behavioural interventions meant most studies were subject to a high-risk of bias, including failure to randomise participation (n = 12), no participant/researcher blinding (n = 11) and lacking a control group (n = 12). At this point in the review, due to the heterogeneity of study designs and outcome measures and high risk of bias, meta-analysis was deemed inappropriate. Instead the decision was made to conduct a narrative synthesis.

## General Overview of Included Studies

Sixteen studies of CBT on refugee children were identified for inclusion. All studies took place since the year 2000, and all were published in journals except for Tsoupas (2011) (39) which was a PHD thesis publication. All studies except for O’Callaghan et al. (2015) and Gomez et al. (2017) occurred in countries of high economic development (40, 41).

The study aims were to treat mental health symptoms of PTSD, anxiety or depression, with most participants having baseline symptoms classed as clinical disease. All studies had either a between-subject or within-subject comparison.

The sample sizes ranged from one participant to eighty-two participants. Most studies included children of mixed ethnicities (39, 42–48), four studies included only children from Africa (33, 47, 49, 50), one study included only children from Yugoslavia (51), one study included only children from Vietnam and Cambodia (52), one study included only children from Syria (41) and one study included only children from Afghanistan (53). Participants with severe mental health symptoms such as psychosis or suicidality were excluded in most studies.

Five of the studies were controlled trials (39, 42, 43, 51), two studies were randomised equivalence trials (40, 49), seven of the study designs were case-series whereby observations on the same group of participants are made before and after the intervention (41, 44–46, 49, 52, 53) and the remaining two were case-reports (50, 54).

The type of CBT was predominantly trauma-focused CBT, and the total duration of therapy ranged from six hours to twenty-eight hours. Seven of the studies used interpreters (39, 41, 44, 44, 45, 49, 52), and in seven studies the parents/care-givers also received therapy (40, 44–47, 49, 50).

Seven of the studies had a follow-up period (40, 42, 43, 46, 49, 50, 52) which ranged from one to twelve months (Tables 1 and 2).

**Table 1:** A general overview of studies (1)

Study	Country	Study Design	Refugee Population	Sample size	Study Aims	Intervention	Measure	Key conclusions
Barrett et al. (2000)	Australia	Controlled before-and-after study	Yugoslavian, all female, mean age 16.3	20	Within subject and between subject comparison of pre/post anxiety symptoms in intervention and control groups	Group CBT	YSR, SCAS,	Significant reduction in anxiety symptoms intervention group, anxiety increased in control group
Ellis et al. (2012)	USA	Case-Series	Somalian, 19 male, 11 females, mean age 13	30	Within subject comparison of pre/post-depression and PTSD symptoms in intervention group	Group and individual CBT	DSRS, PTSD-RI, PWA, WTSS	Non-significant reduction in PTSD and Depression symptoms
Entholt et al. (2005)	UK	Controlled before-and-after study	Mixture ethnicities, 17 male, 9 females, mean age 13	28	Within subject and between subject comparison of pre/post PTSD, depression and anxiety symptoms in intervention and control groups	Group CBT	R-IES, RCMAS, DSRS	Significant reduction in symptoms in those with clinical PTSD
Fox et al. (2005)	USA	Case-series	Vietnamese/ Cambodian, 25 male, 33 females, ages 6–15	58	Within subject comparison of pre/post-depression symptoms in intervention group	Group CBT	CDI	Significant reduction in depression scores
Gormez et al. (2017)	Turkey	Case-series	Syrian, 12 male 20 female, ages 10–15	32	Within subject comparison of pre/post PTSD and anxiety symptoms in intervention group	Group CBT	CPTS-RI, SCAS	Significant reduction PTSD and anxiety symptoms
O’Callaghan et al. (2015)	Congo	Randomised equivalence trial	Congolese, 29 male, 21 females, age 14–17	50	Within subject and between subject comparison of pre/post PTSD symptoms in two intervention groups	Group CBT	UCLA, PTSD-RI. AYPA	Statistically significant improvement PTSD both groups
Ooi et al. (2016)	Australia	Cluster RCT	Mixed ethnicities, 60% African, 53 male, 29 females, age 10–17	82	Within subject and between subject comparison of pre/post PTSD and depression symptoms in intervention and control groups	Group CBT	CRIES-13, DSRS, HSCL-37A, SDQ	Significant reduction in depression, maintained at 3 months. No significant change in PTSD scores

**Table 2:** A general overview of studies (2)

Study	Country	Study Design	Refugee Population	Sample size	Study Aims	Intervention	Measure	Key conclusions
Pfeiffer and Goldbeck (2017)	Germany	Case-series	Afghanistan, all male, aged 14–18	29	Within subject comparison of pre/post PTSD symptoms in intervention group	Group CBT	CATS	Significant reduction PTSD symptoms
Pfeiffer et al. (2018)	Germany	Randomised Controlled Trial	Mixed ethnicities, all unaccompanied young minors, 43 male, 3 females, aged 14–18	99	Within subject and between subject comparison of pre/post PTSD and depression	Trauma Focused CBT	CATS, PHQ8	Significant reduction in PTSD and depression symptoms
Sarkadi et al. (2017)	Sweden	Case Series	Mixed ethnicities, all unaccompanied young minors, 43 male, 3 females, age 14–18	46	Within subject comparison of pre/post PTSD and depression	Trauma Focused CBT	CRIES-8, MADRS-S	Significant reduction in PTSD and depression symptoms
Schottelkorb et al. (2012)	USA	Randomised Equivalency trial	Mixed ethnicities, 17 male, 14 females, age 6–13	31	Within subject and between subject comparison of pre/post PTSD symptoms in two intervention groups	CBT vs child-centred play therapy	UCLA-PTSD, PROPS	Significant reduction in PTSD symptoms following CBT
Tsoupas (2011)	Australia	Controlled before-and-after study	Mixed ethnicity, 12 male, 9 females, aged 7–12	21	Within subject and between subject comparison of pre/post-depression and anxiety symptoms in intervention and control groups	Group and Individual CBT	CDI-S, CSEI, RCMAS	Non-significant reduction in anxiety and depression
Unterhitzenberger et al. (2015)	Germany	Case-series	Mixed ethnicity, 4 male, 2 females, aged 16–18	6	Within subject comparison of pre/post PTSD symptoms in intervention group	Individual CBT	CAPS-CA, PDS	Significant reduction in PTSD symptoms
Unterhitzenberger and Rosner (2016)	Germany	Case-report	African female, aged 17	1	Within subject comparison of pre/post symptoms of PTSD anxiety and depression in individual	Individual CBT	CAPS-CA, UCLA PTSD, CDI, SCARED	Non-significant reduction PTSD, depression and anxiety symptoms maintained at 6 months
Unterhitzenberger et al. (2019)	Germany	Case Series	Mixed ethnicities, all unaccompanied young minors, all male, mean age 17	26	Within subject comparison of pre/post PTSD and depression	Trauma Focused CBT	CATS, MFQ	Significant reduction in PTSD and depression symptoms
Vickers (2005)	UK	Case-report	African, female, aged 14	1	Within subject, pre/post PTSD	Individual CBT	PDS	Non-significant reduction in PTSD symptoms

## Outcomes

Tables 3 and 4 give the statistical outcomes of each study.

**Table 3:** Visual Display of Outcomes Table

Study	Study Type	Study Size	Study Aim	Depression	Anxiety	PTSD	Clinically significant?
Barrett et al. (2000)	Controlled Trial	20	Preventative		↓ *		
Ellis et al. (2012)	Case-series	30	Preventative	↓		↓	
Entholt et al. (2005)	Controlled Trial	28	Preventative	↔	↓	↓ **	
Fox et al. (2005)	Case-Series	58	Preventative	↓ *			
Gormez et al. (2017)	Case-Series	32	Treatment		↓ **	↓ **	
O’Callaghan et al. (2015)	Controlled Trial	50	Treatment	↓ **	↓ **	↓ **	
Ooi et al. (2016)	Controlled Trial	82	Preventative	↓ **		↓	
Pfeiffer et al. (2017)	Case-Series	29	Treatment			↓ **	
Pfeiffer et al. (2018)	Randomised controlled trial	50	Treatment	↓ *		↓ **	
Sarkadi et al. (2017)	Case Series	46	Treatment	↓ * *		↓ *	
Schottelkorb et al. (2012)	Controlled Trial	31	Treatment			↓ **	
Tsoupas (2011)	Controlled Trial	21	Preventative	↓	↓		
Unterhitzenberger et al. (2015)	Case-Series	6	Treatment			↓ * *	
Unterhitzenberger and Rosner (2016)	Case-Study	1	Treatment	↓	↓	↓	
Unterhitzenberger et al. (2019)	Case-Series	26	Treatment	↓ **		↓ *	
Vickers (2005)	Case-Study	1	Treatme Treatment			↓	

In Table 5 the arrows denote the direction of change in the mental health outcome measures following CBT, with a downfacing arrow denoting a reduction in mental health symptoms. The * signifies a statistically significant result whereby p ≤ 0.05 and ** signifies a statistically significant result whereby p ≤ 0.01. Where post-intervention scores became sub-clinical, this was counted as a clinically significant change

**Table 4:** Outcomes (1) (Standard deviation in brackets)

Study	Measure	CBT Pre-intervention mean	CBT Post-intervention mean	Statistically significant?	Control Pre-intervention mean	Control Post-intervention mean	Statistically significant?	Clinically Significant?	Follow up (F/U)
Barrett et al. (2000)	SCAS	39.89 (13.22)	30.43(11.37)	Yes (p < 0.05)	30.64(13.54)	34.20(8.48)	No (p > 0.05)	Anxiety reduced to subclinical	No F/U
Ellis et al. (2012)	DSRS UCLA-PTSD	0.65(0.2) 1.01(0.49)	0.4(0.03) 0.66(0.20)	No (p > 0.05) No(p > 0.05)				Unclear from study	Maintained at 12 months
Entholt et al. (2005)	CRIES-13 DSRS RCMAS SDQ	39.80 (8.40) 12.33(4.7) 16.87(7.22) 9.2(7.76)	33.80 (9.71) 11.67(3.62) 14.67(7.12) 5.40(4.35)	Yes (p = 0.01) No (p = 0.10) No (p = 0.14) Yes (p < 0.05)	38.55(8.37) 12.00(5.37) 16.18(6.57) 6.43(4.69)	42.18(9.38) 13.00(6.57) 18.91(6.04) 5.43(4.28)	No (p = 0.07) No (p > 0.05) No (p = 0.07) No (p > 0.05)	Remained clinical PTSD Remained subclinical anxiety and depression	Not maintained at 2 months
Fox et al. (2005)	CDI	10.38(4.6)	6.15(3.8)	Yes (p < 0.05)				Remained subclinical depression	Maintained at 1 month
Gormez et al. (2017)	SCAS CPTS-RI	53.28(13.78) 23.9(12.76)	40.38(20.59) 17.6(13.64)	Yes (p = 0.01) Yes (p = 0.01)				PTSD reduced to sub-clinical Remained clinical anxiety	None
O’Callaghan et al. (2015)	UCLA-PTSD AYPA	47.77(6.62) 45.08(11.26)	21.54(10.13) 19.08(15.09)	Yes-medium effect size (n2 = 0.55, p < 0.001) Yes- large effect size (n2 = 0.35, p < 0.001)	46.59(7.93) 43.41(12.86)	41.77(9.39) 36.05(10.3)	No (p = 0.057) Yes (p < 0.05)	PTSD, anxiety and depression reduced to subclinical	Not maintained at 6 months
Ooi et al. (2016)	DSRS CRIES-13	10.96 (5.26) 23.02(10.51)	8.68(5.48) 15.88(9.58)	Yes (p < 0.001) Medium effect size No	9.17(4.61) 17.92(11.86)	8.81(4.80) 15.68(8.84)	No (p < 0.05) No (p < 0.05)	Remained subclinical depression and PTSD	Depression improvements maintained at 3 months

Objective 1.) To assess the effects of CBT on PTSD, anxiety and depression in child refugees/AS.

Overall, post-intervention the mental health outcome scores of PTSD, anxiety and depression were lower than the pre-intervention scores for all sixteen studies (See Table 5), suggesting a reduction in mental health symptoms following CBT. This reduction was statistically significant in twelve studies (40–48, 51–53). The Cohen’s d effect sizes (where calculated) were small to large (d = 0.35–1.75, p < 0.05). In eight of the studies, the post-intervention scores became sub-clinical, classed as a “clinically significant” change for the purposes of discussion (40, 41, 44, 46, 47, 50, 51, 54).

**Table 5:** Outcomes (2) (Standard deviation in brackets)

Study	Measure	CBT Pre-intervention mean (SD)	CBT Post-intervention mean (SD)	Statistically significant?	Control Pre-intervention mean (SD)	Control Post-intervention mean (SD)	Statistically significant?	Clinically Significant?	Follow up
Pfeiffer et al. (2017)	CATS	27.6(7.88)	20.7(6.30)	Yes Large effect (d = 0.97, p < 0.001)				Average remained clinical PTSD, 10 children became subclinical	None
Pfeiffer et al. (2018)	-CATS PHQ-8	− 29.97 (1.22) − 11.52(0.71)	− 23.53(1.77) − 8.25 (0.75)	Yes (d = 0.61, p < 0.003), medium effect size Yes (d = 0.67, p < 0.02), medium effect size	-31.85(1.23) − 11.47(0.71)	-30.27(1.73) 11.76(0.76)	No no	No	No
Sarkadi et al. (2017)	-CRIES-8 -MADRS-S	− 29.02(6.33) − 29.26(10.34)	− 25.93(5.96) − 23.39(10.44)	Yes (p < 0.017) Yes (p < 0.001)				No	no
Schottelkorb et al. (2012)	UCLA-PTSD	27.75(6.18)	20.5(13)	Yes (p = 0.01) Large effect (n2 = 0.43, p < 0.001)	27.14(12.16)	17.57(14.09)	Yes (p = 0.01)	PTSD reduced to subclinical	None
Tsoupas (2011)	CDI-S RCMAS	0.82(1.08) 9.18(5.42)	0.73(0.90) 5.73(3.44)	No (p = 0.85) No (p = 0.09)	0.80(0.79) 5.40(2.55)	0.90(1.60) 3.50(3.44)	No (p > 0.05) No (p > 0.05)	Remained with sub-clinical anxiety and depression scores	None
Unterhitzenberger et al. (2015)	CAPS-CA	52 SD not reported	14.5 SD not reported	Yes (p < 0.001)				PTSD reduced to subclinical	None
Unterhitzenberger & Rosner (2016)	CAPS-CA UCLA-PTSD CDI SCARED	50 37 21 14	5 6 16 17	Only 1 individual				PTSD, depression and anxiety reduced to subclinical	Improvement maintained at 6 months
Unterhitzenberger et al. (2019)	-CATS -MFQ	− 30.58 (7.16) − 13.32 (4.26)	− 20.16 (11.63) − 5.63 (4.52)	Yes (p > 0.03) large effect size (d = 1.75) Yes (p < 0.001) large effect size (d = 1.75)				Yes	Yes- immediate, 6 weeks and 6 months
Vickers (2005)	PDS	42	9	Only 1 individual				PTSD scores reduced to subclinical	None

More specifically, the studies measuring PTSD symptoms demonstrated the greatest reduction in symptoms, with nine out of the thirteen studies demonstrating a reduction in symptoms that reached statistical significance (p < 0.05) (40, 41, 43–48, 53) and seven of the studies reaching a higher significance level of p < 0.01 (40, 41, 43, 44, 47, 48, 53). The symptoms became sub-clinical in seven of the studies (40, 41, 44, 46, 47, 50, 54). Where Cohen’s d effect sizes had been calculated they were medium to large (d = 0.61–1.75, p < 0.03) (40, 47, 53).

Of the ten studies which included depression symptoms, six reached statistical significance (p < 0.05) (40, 42, 45, 46, 48, 52) with four reaching a higher statistical significance of p < 0.01 (40, 42, 45, 46). The symptoms became subclinical in three of the studies (33, 34, 45). Where effect sizes were calculated they were small to medium (f = 0.15–5.20) (40, 42).

Of the six studies which included anxiety symptoms, three reached statistical significance (p < 0.05) (40, 41, 51), with two reaching a higher statistical significance of P < 0.01 (40, 41). The symptoms became subclinical in four of the studies (40, 41, 50, 51). Where effect sizes were calculated they were small (f = 0.15) (40).

There were no instances of the intervention group having worse scores post intervention. In contrast, in three of the four studies with control groups the mental health scores worsened over the study period (39, 43, 51).

In five out of seven studies with a follow up period, post-intervention outcome scores were maintained (42, 46, 49, 50, 52). This ranged from one-month to up to a year in Ellis et al. (2012) (49).

Objective 2.) To assess the accessibility and acceptability of CBT for child refugees/AS.

Toupas (2011) found participant satisfaction rates to be as high as 98.2% (39). Barrett et al. (2000) found participant ratings of 4.5/5 for enjoyment and 5/5 for intervention usefulness (51). Additionally, Entholt et al. (2005) and O’shea et al. (2000) found CBT was reported an acceptable intervention to both children and their families (43, 55). The average participant completion rate of the CBT course was 90.8%.

Eight of the studies occurred within a school setting (39, 41–43, 47, 49, 51, 52) and the other eight in a community outpatient setting. Eleven of the studies carried out CBT in groups (39–43, 45, 48, 49, 51–53) and five of the studies delivered individual CBT (44, 46, 47, 50, 54). Five studies used psychology students to carry out the CBT (39, 42, 43, 47, 51), three studies used teachers (40, 41, 52), five used mental health workers (44, 46, 49, 50, 54) and three used social workers (45, 48, 53).

Where non-clinical mental health staff were used, training was received on how to deliver CBT. Training ranged from two to three days and staff were given a CBT session manual to improve intervention fidelity.

## Discussion

Objective 1. To assess the effects of CBT on PTSD, anxiety and depression in child refugees/AS.

This is the first systematic review to focus solely on CBT outcomes in child refugee populations, with promising results. This review demonstrated a universally positive impact of CBT on symptoms of PTSD, depression and anxiety in child refugees across a broad age range, population background, setting and delivery method of CBT.

This review had a greater uniformity of positive results than previous reviews, for example when compared to the mixed positive and negative outcomes of Tyrer and Fazel (2014) (35) and Nocon et al. (2017) (37). This is potentially a consequence of having a narrower research question, for example exploring only CBT outcomes in contrast to the outcomes of a heterogenous group of interventions. These results support the “promising” outcomes of CBT reported in these previous reviews and answer those authors’ calls for further research into CBT outcomes.

The reduction in symptoms brought children into subclinical levels of symptoms in eight of the studies. Furthermore, improvements were greatest in those with the most severe baseline symptoms (45). These findings are in line with those of Foa et al. (56); that those with the highest PTSD symptomology improved the most post-CBT. This is vitally important, given the detrimental effects of mental health symptoms on the physical health, education and acculturation on refugee children as discussed in the introduction (12, 57), and how prompt treatment of mental health symptoms reduces self-harm and suicide (18). This should encourage providers to include children with high symptomology in treatment programmes.

CBT was found to have greatest impact on PTSD scores compared to anxiety and depression. These findings support those of the review by Morina et al. (2017) (34) into child survivors of mass trauma, whereby CBT effect sizes were greatest for PTSD symptoms over depression and anxiety symptoms. This may be a factor of the type of CBT utilised in the studies, as “trauma-focused CBT” was the most common form of CBT used across the studies. This type of CBT focuses on the adaptive processing of past-traumas and related arousal symptoms (16). Further comparative research into the outcomes of different CBT types on specific mental health symptoms would be useful.

Five out of seven studies with a follow-up period found levels of symptoms remained lower than pre-intervention scores, even up to one-year post intervention (49). This contrasts with symptoms of control groups which worsened over the follow-up period. Similar findings have been found in non-refugee children treated with CBT for PTSD following sexual abuse, whereby treatment benefits were found to be sustained at one and two year intervals (58, 59). This suggests that the passage of time alone is not a healing factor for these children. Indeed, in Ooi et al. (2016), post-intervention scores improved further over the follow-up period, suggesting a possible delayed intervention effect (42). A time-lapse between symptom reduction and functional improvement has also been suggested in earlier studies (60, 61) and merits further exploration.

The most common exclusion factor applied within the individual studies was of children with severe mental illness (42, 53). Arguably this excludes some of the most vulnerable in this population, and as discussed earlier potentially those most likely to benefit from CBT. The second most common exclusion was language barriers (42, 43, 53). However, the studies with the resources to use interpreters and trained mental-health workers also reported successful outcomes in children with language barriers and severe symptoms, suggesting such children should not be excluded where possible.

Interestingly, in the two included equivalence studies, the other psychological therapies – “child friendly spaces” and “child centred play therapy” were equally as effective as CBT (40, 47). Therefore, it cannot be ruled out that improvements in symptoms after any psychological therapy are a factor of increased positive adult attention, rather than the specific components of each therapy.

Finally, no adverse effects of CBT were reported. There did not appear to be any evidence of “re-traumatising” following CBT or participants having difficulty understanding the concepts of CBT, concerns previously raised by Rousseau et al. and Kinzie et al. (62, 63).

Objective 2. To assess the accessibility and acceptability of CBT for child refugees/AS.

Most studies were sited in schools, and within the school-timetable for participants, making them accessible. This is in line with the World Health Organization’s (WHO) policy on delivering mental health care through community resources rather than isolated clinics (64). School settings were feasible and associated with high attendance and low attrition rates, with an average intervention completion rate of 90.8% across all studies. This is particularly important given the barriers to accessing mental health care faced by this vulnerable population (22). The success of school-based CBT at reducing symptoms concurs with the previous systematic review by Sullivan and Simonson (2016) (36).

Due to resource limitations and middle-income country settings, seven of the studies trained existing staff members such as teachers and social workers to deliver CBT. This review demonstrates that with additional training, such task-shifting can enable CBT to be delivered feasibly and accessibly in resource-poor settings. Impressively, these studies still gleaned beneficial mental health outcomes with high intervention fidelity.

Furthermore, this review additionally found that teachers were able to successfully identify and refer children struggling with clinical levels of mental health symptoms using the measurement scales. There is potential scope here to strengthen mental health referral processes from schools to specialist services.

The intervention appears acceptable to the children, as high satisfaction and enjoyment rates were reported by the four studies which specifically enquired about them.

Finally, Unterhitzenberger and Rosner (2016) demonstrated how acceptability can be further advanced by using translators as cultural-brokers, to explain cultural differences in emotions and emotional expressions to both the child and the interpreter (50), meriting further exploration.

## Recommendations

This review recommends CBT for the treatment of child refuges with PTSD, depression and anxiety, although acknowledges the research base needs strengthening with more methodologically robust studies. Areas requiring further research include 1.) exploring the effects of different types of CBT on specific mental health disorders and 2.) whether subsequent CBT booster sessions help maintain positive outcomes.

## Limitations

This review has demonstrated a scarcity of primary research on this subject matter. The high risk of bias in several of the included studies should also be considered when interpreting these findings. Furthermore, a lack of language interpretation resources led to the exclusion of potentially relevant foreign-language articles.

## Conclusion

This review is the first to systematically concentrate on the outcomes of CBT on child refugee/AS mental health. CBT consistently reduced symptoms of depression, anxiety and PTSD in child refugees/AS. Results were unanimously positive despite the variety of therapists and settings included. School-based services could circumvent access and financial issues experienced by this population. The training of existing key workers such as teachers and social workers CBT can be a cost-effective and sustainable intervention to help this vulnerable group of patients.

Therefore, this review cautiously recommends its use, yet acknowledges there is a dire need for more methodologically rigorous primary studies of CBT in this population.

## Appendix A

### A: Search Strategies

#### Medline Search Strategy

1 Adolescent/ or child/ or child, preschool/ (2,829,599).

2 Refugees/ (8905).

3 Exp War Exposure/ (404).

4 Asylum seeker.kw. (37).

5 (Asylum adj1 seek$3).ab,ti. (1232).

6 Refugee.ab,kw,ti. (4090).

7 (Expos$3 adj2 war).ab,ti. (400).

8 "Displaced by war".ab,ti. (13).

9 (Internal$2 adj1 displace$4).ab,ti. (474).

10 (War adj2 affected).ab,ti. (236).

11 2 or 3 or 4 or 5 or 6 or 7 or 8 or 9 or 10 (10,959).

12 Cognitive Therapy/ (22,489).

13 Adaptation, psychological/ or exp emotional adjustment/ (89,064).

14 Cognitive behavior therapy.kw. (249).

15 Cognitive therapy.kw. (225).

16 (Cognitive adj2 therap$3).ab,ti. (14,228).

17 12 or 13 or 14 or 15 or 16 (113,528).

18 1 and 11 and 17 (260).

#### Embase Search Strategy

1 Preschool child/ or adolescent/ or child/ (2,446,671).

2 Refugees/(10,687).

3 Exp War Exposure/ (186).

4 Asylum seeker.kw. (89).

5 (Asylum adj1 seek$3).ab,ti. (1756).

6 Refugee.ab,kw,ti. (5537).

7 (Expos$3 adj2 war).ab,ti. (526).

8 "Displaced by war".ab,ti. (13).

9 (Internal$2 adj1 displace$4).ab,ti. (698).

10 (War adj2 affected).ab,ti. (310).

11 2 or 3 or 4 or 5 or 6 or 7 or 8 or 9 or 10 (13,790).

12 Cognitive Therapy/ (42,674).

13 Psychological adjustment/ or coping behavior/ (52,249).

14 Cognitive behavior therapy.kw. (1185).

15 Cognitive therapy.kw. (1526).

16 (Cognitive adj2 therap$3).ab,ti. (25,252).

17 12 or 13 or 14 or 15 or 16 (100,233).

18 1 and 11 and 17 (139).

#### PsycINFO Search Strategy

1 Refugees/ (5254).

2 Exp War Exposure/ (0).

3 Asylum seeker.kw. (0).

4 (Asylum adj1 seek$3).ab,ti. (1289).

5 Refugee.ab,kw,ti. (4234).

6 (Expos$3 adj2 war).ab,ti. (549).

7 "Displaced by war".ab,ti. (14).

8 (Internal$2 adj1 displace$4).ab,ti. (292).

9 (War adj2 affected).ab,ti. (375).

10 1 or 2 or 3 or 4 or 5 or 6 or 7 or 8 or 9 (7812).

11 Cognitive Therapy/ (13,107).

12 Adaptation, psychological/ or exp emotional adjustment/ (21,001).

13 Cognitive behavior therapy.kw. (0).

14 Cognitive therapy.kw. (0).

15 (Cognitive adj2 therap$3).ab,ti. (26,343).

16 11 or 12 or 13 or 14 or 15 (52,686).

17 Child.mp. (322,109).

18 Children.mp. (498,343).

19 Adolescent.mp. (153,423).

20 Adolescence.mp. [mp = title, abstract, heading word, table of contents, key concepts, original title, tests & measures] (67,089).

21 17 or 18 or 19 or 20 (741,618).

22 10 and 16 and 21 (83).

#### Cochrane Search Strategy

1 Adolescent/ or child/ or child, preschool/(122,082).

2 Refugees/ (89).

3 Exp War Exposure/ (0).

4 Asylum seeker.kw. (4).

5 (Asylum adj1 seek$3).ab,ti. (23).

6 Refugee.ab,kw,ti. (158).

7 (Expos$3 adj2 war).ab,ti. (29).

8 "Displaced by war".ab,ti. (1).

9 (Internal$2 adj1 displace$4).ab,ti. (19).

10 (War adj2 affected).ab,ti. (37).

11 2 or 3 or 4 or 5 or 6 or 7 or 8 or 9 or 10 (269).

12 Cognitive Therapy/ (6993).

13 Adaptation, psychological/ or exp emotional adjustment/ (3863).

14 Cognitive behavior therapy.kw. (20).

15 Cognitive therapy.kw. (2432).

16 (Cognitive adj2 therap$3).ab,ti. (9655).

17 12 or 13 or 14 or 15 or 16 (16,593).

18 1 and 11 and 17 (17).
